# Self-expandable metal stent (SEMS) placement for malignant colonic obstruction in palliative patients: post-procedural morbidity and long-term outcomes

**DOI:** 10.1007/s10151-026-03332-6

**Published:** 2026-05-13

**Authors:** M. Serrano-Navidad, E. Kreisler, E. Alba, S. Biondo

**Affiliations:** 1 Colorectal Unit, Department of General and Digestive Surgery, Bellvitge University Hospital, C/Feixa Llarga S/N, L’Hospitalet de Llobregat, 08907 Barcelona, Spain; 2 IDIBELL (Bellvitge Biomedical Investigation Institute), Barcelona, Spain; 3 Interventional Radiology Unit, Department of Radiology, Bellvitge University Hospital, C/Feixa Llarga S/N, L’Hospitalet de Llobregat, 08907 Barcelona, Spain; 4 Departament de Ciències Clíniques, Facultat de Medicina I Ciències de La Salut, Universitat de Barcelona (UB), C/Feixa Llarga, S/N, 08907 L’Hospitalet de Llobregat, Spain

**Keywords:** Self-expandable metal stent, Colon obstruction, Palliative, Morbidity, Mortality, Complications, Survival, Reintervention

## Abstract

**Background:**

Self-expandable metal stents (SEMS) are recommended as a first-line treatment for malignant colonic obstruction in palliative patients. However, published outcomes remain controversial. This study aimed to evaluate the morbidity associated with SEMS placement in patients with tumor-related colonic obstruction in a palliative setting. Secondary objectives included long-term outcomes and the impact on subsequent oncological treatment.

**Methods:**

A retrospective study was conducted on 146 consecutive palliative oncological patients treated at a tertiary center between 2008 and 2023. Patients were categorized into three groups based on the cause of palliation: unresectable stage IV colon cancer (Group I), colon cancer with comorbidities and poor performance status (Group II), and extrinsic compression from extracolonic malignancies (Group III).

**Results:**

Clinical resolution of obstruction was achieved in > 80% of patients, with significant differences among groups (88% in Group I, 79% in Group II, and 59% in Group III; *p* = 0.009). Early morbidity occurred in 38% of cases and severe complications (≥ Clavien-Dindo III) in 31%. Morbidity was more frequent in Group III (65%). Thirty-day mortality was 15%. Only 12% of patients resumed chemotherapy after SEMS placement, with a median delay of 34 days. Median overall survival after SEMS placement was 4 months. Over 95% of patients died because of disease progression.

**Conclusions:**

SEMS placement in palliative patients is effective in relieving obstruction but carries a high risk of complications, especially in extracolonic malignancies. Less than a third of patients continue oncological treatment after stenting, highlighting the need for careful patient selection.

## Introduction

Colonic obstruction in palliative patients constitutes a significant clinical challenge, particularly in those with advanced malignancies and severe comorbidities. Endoscopic stent placement has emerged as a minimally invasive alternative to surgical intervention, aimed at relieving obstruction and improving quality of life. In advanced colonic cancer, approximately 8–13% of patients present with large-bowel obstruction and need urgent measures to relieve symptoms and prevent life-threatening complications such as bowel perforation, ischemia, and sepsis [1]. For years, emergency surgery, including resection or derivative stoma, has been the standard approach for managing colonic obstruction of tumoral origin. Nevertheless, surgical interventions are often associated with high short-term morbidity and mortality, particularly in patients with advanced disease and limited life expectancy [2]. Consequently, self-expandable metal stents (SEMS) have emerged in the last few decades as a viable alternative for palliative treatment, offering a less invasive technique to alleviate obstructive symptoms and avoid the need for emergency surgery in patients with limited survival prospects [3].

Following its first description by Dohmoto in 1991 for treating colon obstruction in palliative patients [4], SEMS placement has been increasingly recognized as a preferred palliative strategy for colonic obstruction in patients with advanced malignancies who are not candidates for curative resection. The European Society of Gastrointestinal Endoscopy (ESGE) updated its guidelines in 2020, recommending SEMS placement as a first-line palliative approach in patients with non-resectable obstructing colonic and extracolonic malignancies [1], with a lower initial complication rate and faster symptom resolution compared with surgical diversion [5], avoiding permanent stomas, which can significantly enhance their quality of life. A systematic review and meta-analysis by Veld et al. (2020) [6] comparing SEMS with emergency surgery found that stenting was associated with a higher technical success rate, fewer perioperative complications, and shorter recovery times. Studies have reported varying rates of stent failure, with some estimating that repeat interventions are needed in up to 30% of patients [7]. Early morbidity of 13.6% and late morbidity of 23.2% have been described, as well as mortality of 3.9–6.3% [3, 4]. Furthermore, patients with peritoneal carcinomatosis or extensive tumor burden may experience limited symptom relief following SEMS placement, necessitating a careful selection process to identify appropriate candidates.

Debates persist regarding the long-term effectiveness, safety profile, and optimal patient selection for SEMS placement in the palliative setting. Stent-related adverse events, including migration, re-obstruction due to tumor ingrowth, and perforation, remain significant concerns. According to our previous systematic review, given the various alternatives and the lack of high-quality evidence, the treatment of distal colonic obstruction should be tailored to each patient [8].

This study aimed to evaluate the post-procedure morbidity after placing an SEMS for colonic obstruction in oncological palliative patients. Short- and long-term complications, overall survival (OS) after initial treatment, change in performance status, and the rate of patients who were candidates for chemotherapy and successfully restarted the chemotherapy were analyzed as secondary outcomes.

## Materials and methods

### Study population

A retrospective study was conducted within an internal prospective cohort of consecutive oncological patients who presented with colonic obstruction and underwent SEMS with palliative intention between 2008 and 2023 at Bellvitge University Hospital in Barcelona, Spain. Patients with a colon obstruction of tumoral origin located distal or adjacent to the splenic flexure were included in the study. The location of the obstruction was classified as proximal when it was in the transverse colon or adjacent to the splenic flexure and as distal when the obstruction was located distal to the splenic flexure. Exclusion criteria included suspected colonic perforation, as well as lesions localized within the right colon or the mid-to-lower rectum. Three groups of palliative patients with colonic obstruction were defined depending on the cause for which they were considered palliative: group I, comprised of patients with unresectable stage IV colon cancer, defined as distant metastasis (excluding resectable hepatic or extra-hepatic metastases) diagnosed by CT scan; group II, comprised of patients with colon cancer with severe comorbidities or low-performance status that contraindicated further surgical treatment; group III, comprised of patients with extrinsic compression by tumor implantation in palliative patients due to a non-colonic origin cancer.

Malignant colonic obstruction was defined as the total absence of gases and/or feces during at least the past 24 h, associated with abdominal distension, with or without nausea and vomiting, and an abdominal x-ray showing dilated colon, caused by a colonic or extracolonic neoplastic infiltration. The diagnosis was confirmed using a Gastrografin^®^ enema or an abdominal CT scan with intravenous contrast.

The SEMS placement was performed under conscious sedation by interventional radiologists in the Interventional Radiology Unit, as described in our previous article [9]. Tumor localization was achieved by administering a Gastrografin enema with fluoroscopic guidance, facilitating advancement of the catheter to the stenotic site. The enema tube serves as the primary access point for the procedural materials, utilizing a methodology analogous to vascular access techniques: initial puncture with an Abbocath Teflon needle (Hospira Venisystems, Lake Forest, IL, USA), followed by insertion of a Terumo hydrophilic guidewire (Terumo Co., Tokyo 151–0072, Japan) to facilitate the placement of the 7F CORDES introducer (CORDES Co., FL, USA), advancement of the guide and a catheter until it successfully traverses the obstruction, exchange the hydrophilic wire for a rigid Amplatz Super Staff guide (Boston Scientific, FL 33166, USA), removal of the initial catheter and introducer, passage of the catheter with the uncovered Wallflex Enteral® stent (Boston Scientific, Natick MA 01760–1537, USA) through the rigid guide, and finally release of the stent, available in lengths of 6, 9, or 12 cm, always ensuring a 1–2-cm extension beyond the proximal and distal margins of the stenosis, and a 25 or 30 mm diameter, ensuring it is precisely centered within the stenosis. Patients without correct stent placement through the stenosis during the procedure were not included in this study. Clinical success was defined as clinical evidence of obstruction resolution within 24 h of stenting, confirmed radiologically, with no complications related to stent insertion (absence of decompression and colon perforation). Failure of stenting treatment included: lack of decompression after stent insertion, leading to immediate obstruction needing endoscopic or surgical intervention (early failure), and obstruction after initial successful decompression due to tumor progression (late failure).

Demographic variables were recorded, including the Charlson index [10], performance status measured with the Barthel index [11], which measures a patient's ability to perform basic activities of daily living, ECOG score [12], which assesses a patient's level of functioning in terms of self-care and daily activity, and Karnofsky scale [13], which evaluates a patient's overall functional status and ability to carry out normal activities. The main study variables were the procedure success and post- procedure morbidity after stent placement. Complications were classified according to the Clavien-Dindo score [14]. Treatment following complications (including the need for surgical treatment), chemotherapy after stent placement, early morbidity, and mortality were analyzed. Early morbidity was defined as morbidity within 30 days following the procedure. The study protocol was reviewed and approved by the institutional research and ethics board.

### Statistical analysis

For the analysis, the patients were divided into three groups described above. Quantitative variables that followed a normal distribution were measured by mean and standard deviation, whereas those that did not follow a normal distribution were measured by median and interquartile range. Qualitative variables were described using frequencies and percentages. For comparison between group means, ANOVA was used for continuous variables and the chi-square test for categorical variables. To compare variables before and after stent placement, the Wilcoxon test was employed. To investigate the interactions between measurements and study factors, multinomial regression models with stepwise selection, logistic regression with stepwise selection, and survival analysis (Kaplan-Meier curves and Cox proportional hazards models with stepwise logistic regression for covariate selection) were performed. Results were expressed as odds ratios (ORs) with 95% confidence intervals (CIs). RStudio (version 4.3.1; R Project for Statistical Computing, Vienna, Austria) was used to manage patient data and to perform statistical analysis. A *p*-value < 0.05 was considered statistically significant.

## Results

The demographic characteristics of the total sample and the three groups are described in Table 1. A total of 146 palliative patients were included in the analysis. Groups I, II, and III consisted of 94 (64%), 35 (24%), and 17 (12%) patients, respectively. The mean age was significantly higher in Group II (*p* < 0.001). Men were more prevalent, with no significant differences between groups. The mean Charlson index was > 8 in all groups, mainly due to severe comorbidities or metastatic cancer.

**Table 1:** Characteristics of palliative patients

	Overall N = 146 (%)	I N = 94 (%)	II N = 35 (%)	III N = 17 (%)	*P*-value
Age (years)	70 (14)	67 (13)	82 (10)	69 (12)	< 0.001
Gender Male Female	86 (59) 60 (41)	57 (61) 37 (39)	22 (63) 13 (37)	7 (41) 10 (59)	0.279
Arterial hypertension	41 (28)	20 (21)	17 (49)	4 (24)	0.008
Diabetes mellitus	40 (27)	23 (24)	15 (43)	2 (12)	0.035
Ischemic cardiomyopathy	21 (14)	6 (6.4)	14 (40)	1 (5.9)	< 0.001
Heart failure	27 (18)	9 (9.6)	16 (46)	2 (12)	< 0.001
Peripheral vascular disease	3 (2.1)	2 (2.1)	1 (2.9)	0 (0)	0.790
Stroke	4 (2.7)	1 (1.1)	3 (8.6)	0 (0)	0.051
Hemiplegia	1 (0.7)	0 (0)	1 (2.9)	0 (0)	0.203
Dementia	9 (6.2)	0 (0)	9 (26)	0 (0)	< 0.001
COPD	31 (21)	17 (18)	12 (34)	2 (12)	0.081
CKD	25 (17)	11 (12)	10 (29)	4 (24)	0.059
Connective tissue disease	3 (2.1)	2 (2.1)	1 (2.9)	0 (0)	0.790
Ulcer	7 (4.8)	3 (3.2)	3 (8.6)	1 (5.9)	0.435
Liver cirrhosis	7 (4.8)	1 (1.1)	5 (14)	1 (5.9)	0.007
Metastatic disease	116 (79)	94 (100)	10 (29)	12 (71)	< 0.001
Peritoneal disease	29 (20)	25 (27)	3 (8.6)	1 (5.9)	0.023
Leukemia	2 (1.4)	2 (2.1)	0 (0)	0 (0)	0.571
Lymphoma	1 (0.7)	1 (1.1)	0 (0)	0 (0)	0.757
Charlson	9.3 (2.1)	9.3 (1.9)	9.8 (2.1)	8.3 (2.9)	0.054
Barthel (initial)	84 (23)	95 (11)	66 (22)	78 (39)	< 0.001
Barthel (after stent)	51 (26)	58 (26)	37 (22)	70 (12)	0.008
Karnofsky (initial)	84 (12)	87.5 (80.1–94.9)	80.0 (74.0–91.2)	81.67 (69.4–93.9)	0.445
Karnofsky (after stent)	67 (15)	65.3 (53.9–76.7)	46.7 (32.3–61.0)	70.0 (27.0–93.0)	0.268
ECOG (initial)					
ECOG < 2	23 (68)	19 (83)	1 (14)	3 (75)	0.003
ECOG ≥ 2	11 (32)	4 (17)	6 (86)	1 (25)	
ECOG (after stent)					
ECOG < 2	12 (30)	8 (31)	0 (0)	4 (80)	0.007
ECOG ≥ 2	28 (70)	18 (69)	9 (100)	1 (20)	
ASA (initial)					
ASA 1–2	9 (53)	3 (50)	2 (29)	4 (100)	0.073
ASA ≥ 3	8 (47)	3 (50)	5 (71)	0 (0)	
Obstruction localization					
Distal	117 (87)	78 (88)	29 (85)	10 (91)	0.877
Proximal	17 (13)	11 (12)	5 (15)	1 (9.1)	
Stent as initial treatment	77 (53)	38 (40)	31 (89)	8 (47)	< 0.001

*ASA* American Society of Anesthesiologists*, COPD* chronic obstructive pulmonary disease*, CKD* chronic kidney disease

Group II had a statistically significantly higher prevalence of serious conditions such as cardiovascular risk factors and heart disease or liver cirrhosis, but a lesser proportion of metastatic disease. The initial Barthel index and ECOG scores were worse in this group, since these patients exhibited significant functional impairment. In contrast, Group I had the best initial Barthel Index and ECOG performance status scores.

In 87% of the patients, the stent was placed distal to the splenic flexure. The most frequent location of the obstruction was in the sigmoid colon and recto-sigmoid junction. In patients considered palliative because of significant comorbidities (group II), the initial treatment was stenting in 89% of cases.

Colonic obstruction clinically resolved in 82% (120/146) of the patients, being significantly different between groups: 88% (83/94) in group I, 79% (27/35) in group II, and only 59% (10/17) in group III (*p* = 0.009) (Table 2). Stent placements in group III patients were less likely to be successful than in group I patients. Overall, the early morbidity rate was 38%, and this was greater in extracolonic malignancies than in the colon cancer groups. Complication rates ≥ III Clavien-Dindo were 31%: perforation (7.5%) and migration (4.8%) were the most frequent in the short term, while late failure occurred in 8.9% of patients. In group III, severe complications ≥ III Clavien-Dindo were more frequent than in other groups (59%, *p* = 0.028), with perforation (12%) and late failure (18%) being more prevalent (*p* = 0.044).

**Table 2:** Outcomes after stent placement

	Overall N = 146 (%)	I N = 94 (%)	II N = 35 (%)	III N = 17 (%)	*P*-value
Clinical success rate	120 (82)	83 (88)	27 (79)	10 (59)	0.009
30-day morbidity	55 (38)	37 (39)	7 (20)	11 (65)	0.007
30-day mortality	22 (15)	11 (12)	7 (21)	4 (24)	0.276
Clavien-Dindo					
< III	19 (13)	11 (12)	5 (14)	3 (18)	0.028
≥ III	45 (31)	26 (28)	9 (26)	10 (59)	
Complications					
Perforation	11 (7.5)	8 (8.5)	1 (2.9)	2 (12)	0.044
Migration	7 (4.8)	5 (5.3)	1 (2.9)	1 (5.9)	
Hemorrhage	2 (1.4)	2 (2.1)	0 (0)	0 (0)	
Late failure	13 (8.9)	7 (7.4)	3 (8.6)	3 (18)	
Other	4 (2.7)	4 (4.3)	0 (0)	0 (0)	

**Fig. 1:**
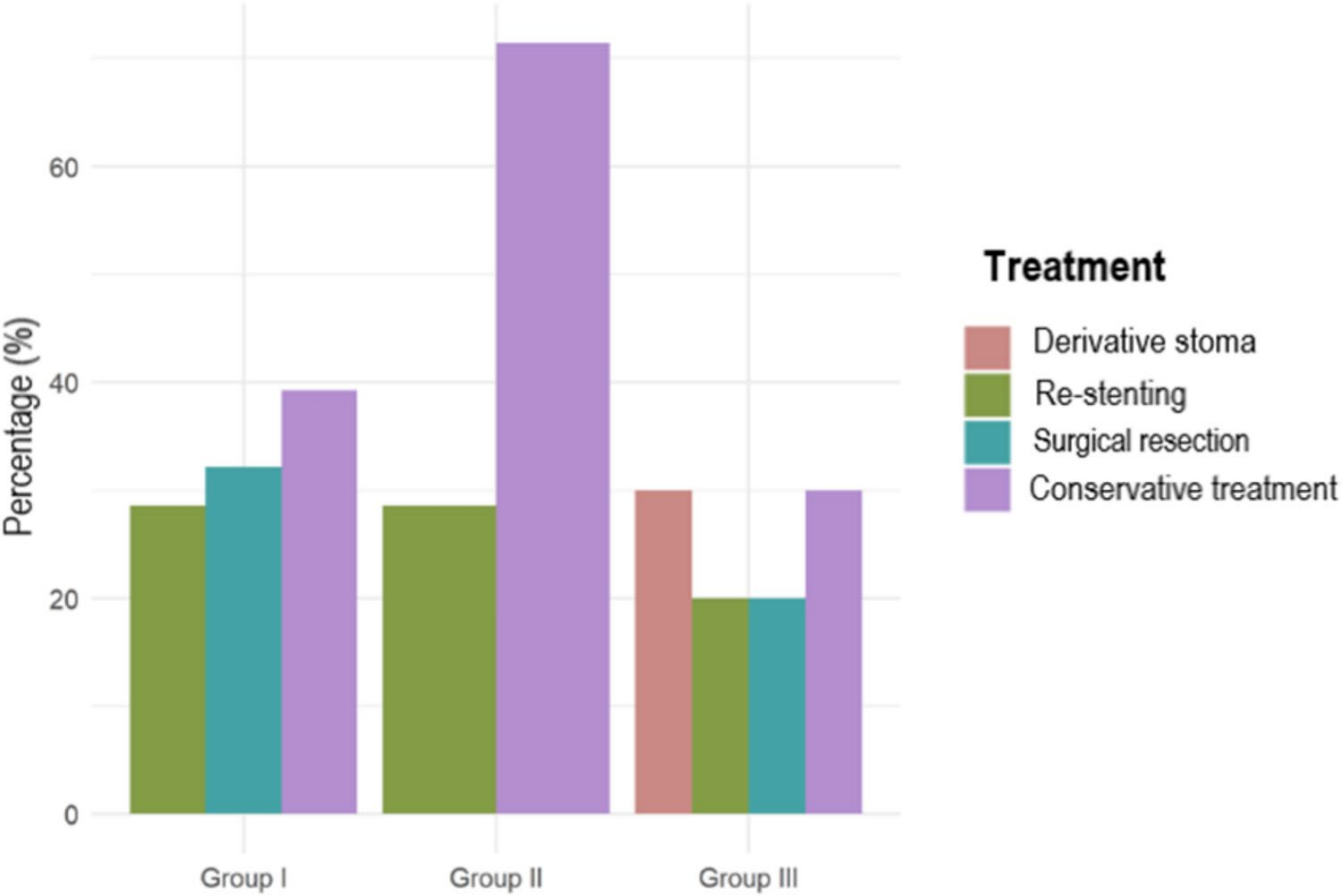
Treatment of the complications per group

**Fig. 2:**
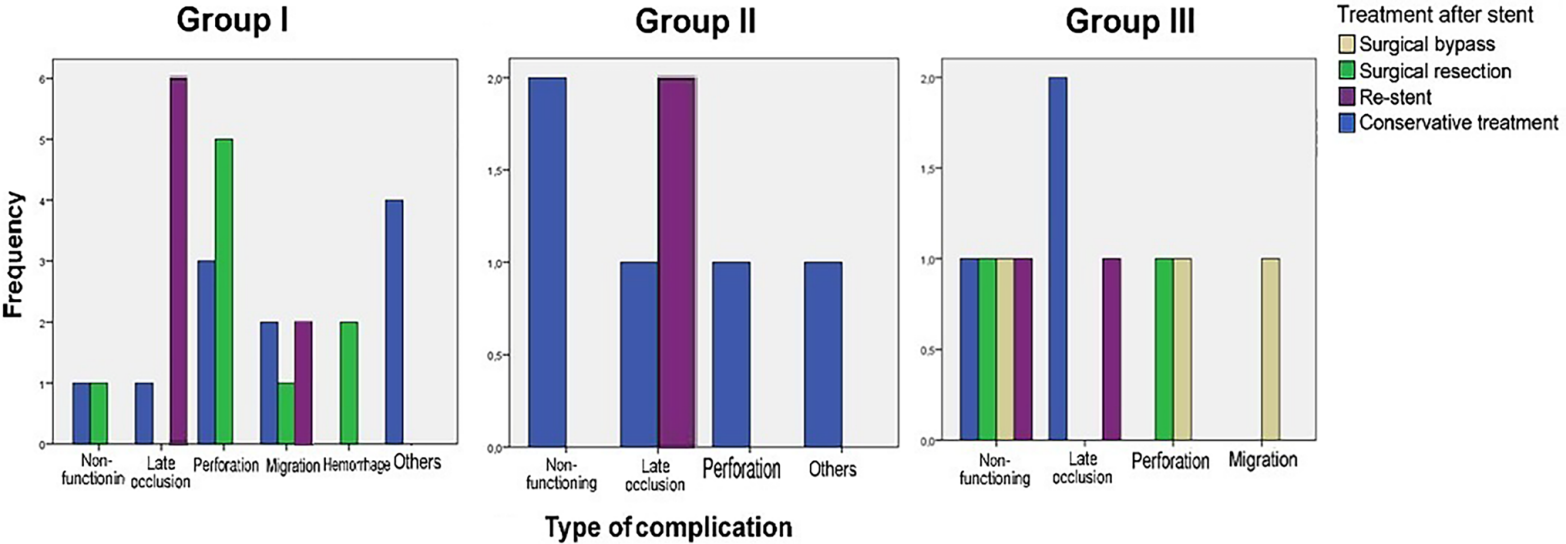
Treatment of each complication per group

In total, 40 patients (31%) required urgent surgery because of stent-related complications, and 14 patients (9.6%) required the placement of a new stent. Many of the complications following stent placement were managed conservatively (Fig. 1). Performance status decreased statistically across all three classifications (Barthel, Karnofsky, and ECOG) after stent placement (Table 3). In the short term, patients in Groups I and III who developed perforation were primarily managed with surgical resection or the creation of a diverting stoma, whereas stent migration was addressed by either stent replacement or surgical intervention. In contrast, short-term complications in Group II were predominantly managed conservatively, with re-stenting employed less frequently. When late failure occurred, a  stent replacement was mainly performed in group I and II, while in groupIII, conservative treatment was preferred (Fig. 2).

**Table 3:** Barthel, Karnofsky, and ECOG before and after stent placement in all groups

	Initial N = 146^1^	After stent N = 94^1^	*P*-value
Barthel	70 (50)	50 (35)	0.001
Karnofsky	85 (15)	60 (30)	0.041
ECOG			
< 2	19 (68)	12 (30)	0.004
≥ 2	9 (32)	28 (70)

**Fig. 3:**
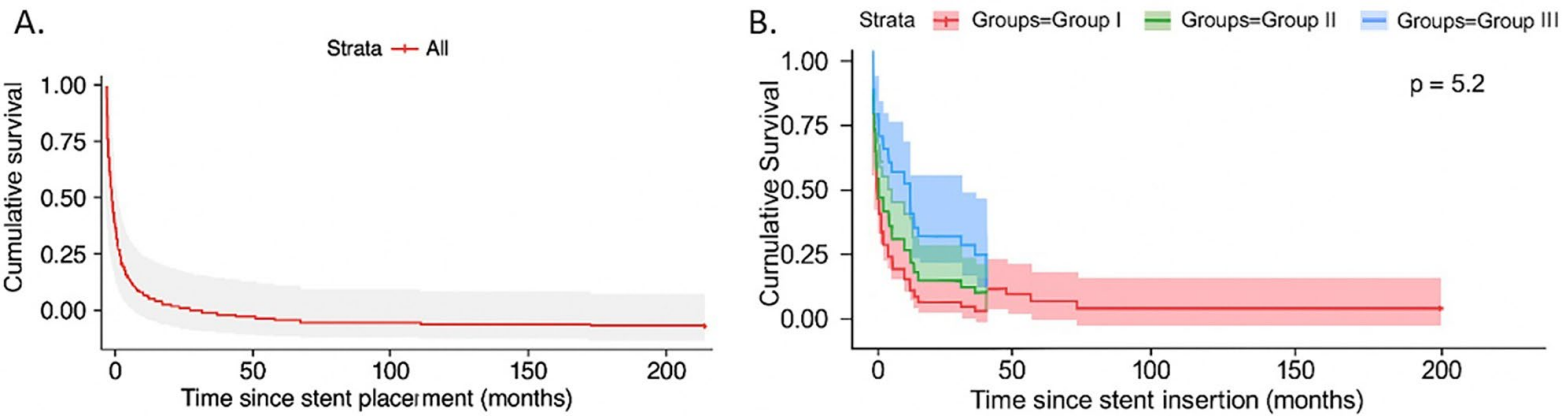
Survival functions since stent placement. **A** all groups, **B** per group

Before the obstructive episode, 66% of patients in group I, 2.9% in group II, and 58.8% in group III (*p* < 0.001) received chemotherapy. After stent placement, only 12% received oncological treatment, with a mean time of 34 (22–70) days until reinitiation of chemotherapy. Only 22.5% of patients in group I and 29.9% in group III were able to resume their oncological treatment (Table 4). The likelihood of resuming chemotherapy was reduced by 42% in patients with higher Charlson comorbidity scores (OR = 0.58; 95% CI: 0.33–0.96; *p* = 0.043). Mortality at 30 days rose to 15%, without statistically significant differences among the groups. The highest 30-day morbidity (65%) and 30-day mortality (24%) were found in group III. The estimated median overall survival after stent placement was 4 months, and non-statistical differences were observed between groups I–II (4 months) and group III (3 months) (*p* > 0.05) (Fig. 3). Clinical success in resolving the occlusion was associated with a reduction in the risk of death (HR = 0.02; 95% CI 0.01–0.06, *p* < 0.001). A total of 96% of the patients died because of progression of their oncological disease. (Table 5).

**Table 4:** Chemotherapy treatment per group

	Overall N = 146 (%)	I N = 94 (%)	II N = 35 (%)	III N = 17 (%)	*P*-value
QMT (any time)^*1*^	73 (50)	62 (66)	1 (2.9)	10 (59)	< 0.001
QMT after stent placement^*1*^	18 (12)	14 (15)	1 (2.9)	3 (18)	
QMT after stent placement/QMT any time		22.5%	100%	29.9%	
Days until treatment restarted^*2*^	34 (22–70)	34 (22–70)	48	27 (12–103)	0.989

^1^*n* (%), ^2^median (IQR)

**Table 5:** Follow-up after the stent placement

	Overall N = 146 (%)	I N = 94 (%)	II N = 35 (%)	III N = 17 (%)	*P*-value
Mean OS after stent placement (months)^*1*^	11 (7.4–15)	13 (6.8–18)	7 (3.9–11)	13 (4.6–21)	0.523
Median OS after stent placement (months)^*2*^	4 (1–13)	4 (1–12)	4 (0–12)	3 (0–14)	0.290
Mean OS after initial treatment (months)^*1*^	19 (13–24)	18 (12–25)	9.8 (5.7–14)	35 (7.8–61)	0.023
Median OS after initial treatment (months)^*2*^	10 (3–20)	10 (3–20)	6 (1–17)	14 (2–48)	0.240
Disease progression as a cause of death^*3*^	137 (96)	89 (98)	32 (91)	16 (94)	0.260

*OS* overall survival

^1^Mean (SD), ^2^Median (IQR), ^3^n (%)

## Discussion

Our study shows how the use of SEMS in patients with advanced cancer in a palliative setting has become an essential tool in the management of malignant acute colonic obstruction, particularly in patients with significant comorbidities, in whom SEMS is the first-line treatment option in nearly 90% of cases, mainly because the benefits of surgery remain controversial. However, despite the favorable outcomes for obstruction resolution, our series highlights that in this context, the complication rate is high, > 30%, and long-term survival is limited to a median of 4 months. Therefore, as emphasized by Cardoso & Rodrigues-Pinto (2025) [7] in a recent review on the use of SEMS as a palliative measure, appropriate patient selection is crucial. These findings emphasize the importance of individualized treatment decisions based on comorbidities and prognosis.

The definition of palliative patients varies substantially across studies. Therefore, a more precise characterization is required, specifying the reasons for classifying patients as palliative. Such a definition should account not only for the underlying oncological disease but also for comorbidities, which are frequently incorporated into clinical decision-making when determining the most appropriate treatment strategy. Our study included 146 patients, with Group I (colon cancer) comprising 64%, Group II (severe comorbidities) 24%, and Group III (extracolonic malignancies) 12%. This analysis provides stronger evidence on the impact of SEMS in terms of complications, survival, and the likelihood of restarting oncological treatment according to the palliative reason, which will help assess treatment expectations for malignant colonic obstruction based on patient characteristics. Group II patients were older and exhibited worse functional status (Barthel and ECOG scores), along with a higher prevalence of cardiovascular and hepatic conditions. Group III showed the lowest clinical success rate in resolving obstruction (59%) and the highest rate of severe complications (≥ III Clavien-Dindo), including perforation and late failure. These findings align with existing literature [15], indicating that colonic stenting is effective in managing obstruction in advanced colorectal cancer but less successful in extracolonic malignancies. The increased complication rates in Group III are consistent with previous reports highlighting poor outcomes in non-colorectal obstructions [16, 17]. Our analysis of the cause of the patients' palliative situation may help to weigh the benefits and drawbacks of SEMS placement regarding morbidity.

There are notable discrepancies among studies regarding the effectiveness of SEMS as a palliative treatment for malignant colonic obstruction, particularly in symptom relief, complication rates, and overall survival. The ESGE 2020 guidelines recommend SEMS as a palliative option for patients with unresectable colonic or extracolonic malignancies causing obstruction, citing a technical success rate of approximately 96% and a clinical success rate of around 92% [1]. Our study had a clinical success rate of 82% for SEMS placement in patients with malignant colonic obstruction, which increased to 88% in cases of colorectal cancer with unresectable metastases and dropped below 60% in cases where the obstruction was due to extrinsic compression from extracolonic malignancies. This finding aligns with existing literature, which reports clinical success rates for SEMS placement in extrinsic obstructions ranging from 25% to 87.5%, often lower than those observed in intrinsic colorectal obstructions [18, 19]. The reasons for this can be attributed to differences in tumor biology and, frequently, to more extensive intestinal involvement, which may render the stent insufficient to resolve the obstructive condition and lead to a higher rate of complications.

Van den Berg et al. (2015) [5] reported that SEMS placement in a palliative setting yielded a median OS of 121 days, with 82% of patients avoiding the need for further surgical intervention. However, re-obstruction occurred in 23% of cases, necessitating additional procedures. Although nearly one-third of patients required urgent surgery for stent-related complications, and nearly 10% required re-stenting, the median overall survival (OS) in our study was 4 months, consistent with previously published data [6]. Mortality following the procedure in our sample was also significant, although no meaningful differences were observed between the groups. Thirty-day mortality was 15%, with Group III showing the highest morbidity and mortality. Successful resolution of obstruction correlated with reduced mortality risk, which may not reflect an unfavorable outcome of the SEMS per se, but rather its long-term efficacy, highly influenced by tumor progression and stent patency, as well as the poor prognosis of these patients when faced with a severe complication such as intestinal obstruction. The association between clinical success and improved survival reinforces the importance of technical efficacy in palliative interventions.

Variability in technicalities across different centers and in operator expertise remains a concern and can be a challenge when reaching conclusions. Most single-center studies have small patient numbers, and those with larger sample sizes are multicenter studies with high variability in technical details across centers and operator expertise. Variations in stent types, placement techniques, and post-procedure care can introduce additional biases, making it challenging to draw definitive conclusions about the procedure’s success or complications. Our study provides a larger sample of patients within a standardized protocol for stent placement with long-term follow-up, which will undoubtedly contribute to increasing the evidence base for SEMS placement in palliative cases.

Published studies examining the resumption of chemotherapy treatment after SEMS placement have reported mixed results. A multicenter study by Cézé et al. (2016) [20] assessed the safety of combining SEMS with systemic chemotherapy in advanced colorectal cancer, revealing an increased risk of perforation in patients receiving bevacizumab-based regimens. Consequently, the decision to place a SEMS should consider the planned oncological treatment to mitigate potential complications. In our experience, the deterioration in performance status was straightforward, and only 12% of patients restarted oncological treatment after stent placement, compared with less than a third of the patients receiving chemotherapy before stent placement. This low rate is fundamentally linked to the advanced frailty and palliative status of this population, rather than the success of the SEMS placement. It is imperative to clarify that the primary limiting factor was the patients' clinical baseline at the time of obstruction. Most of the patients presented with a deteriorated performance status or a high burden of comorbidities, which, when compounded by the acute obstructive event, rendered them ineligible for further cytotoxic treatment. Furthermore, all patients were already categorized as terminal because of the progression of their primary malignancy or synchronous metastatic disease. In these instances, the stenting procedure is strictly a salvage intervention aimed at symptom relief and avoiding emergency surgery, rather than a bridge to further systemic therapy. Finally, the occurrence of intercurrent complications frequently hindered any therapeutic escalation. Consequently, the inability to resume chemotherapy reflects the natural history of late-stage oncological disease in a palliative setting, where the clinical priority shifts from prolonging survival to maintaining intestinal patency and quality of life, as documented in similar studies [21–23].

As advancements in stent technology and multidisciplinary management continue, SEMS is likely to remain a cornerstone of palliative care for malignant colonic obstruction. Current evidence supports its use as a first-line palliative strategy, but continued research is needed to optimize patient outcomes and refine clinical guidelines. Mauro et al. (2024) [24] emphasized the role of a multidisciplinary approach in optimizing outcomes, noting that SEMS placement should be considered within a broader treatment plan that incorporates oncological therapies. Incorporating the underlying cause of a patient's palliative condition, as established in our study, can significantly enhance shared decision-making processes—not only among healthcare professionals but also between clinicians and patients.

Strengths of this study include a robust sample size, well-defined patient classification, and a comprehensive evaluation of functional status and complications. However, the retrospective design limits causal inference, and the criteria for stent placement were not explicitly described. Moreover, the low rate of chemotherapy resumption may have been influenced by unmeasured clinical or patient-related factors. Selection bias could have contributed to more favorable outcomes reported in other retrospective series on the same topic. Finally, sampling error cannot be excluded, as evidenced by the wide ranges observed in the 95% confidence intervals.

Despite the growing acceptance of SEMS for the management of palliative colonic obstruction, several challenges persist. The role of novel stent designs and biodegradable stents in reducing complications such as migration and re-obstruction warrants further investigation. The use of patient-reported outcome measures (PROMs) in routine clinical practice to consider not only the morbidity and mortality associated with SEMS but also the patient's personal values and care goals will undoubtedly help provide quality treatment to these patients in the final stage of their oncological process.

## Conclusions

Endoscopic stenting is a viable option for palliation in patients with colonic obstruction, particularly in those with colorectal cancer. Patients with severe comorbidities or extracolonic malignancies face higher risks and lower therapeutic benefits. Clinical success in resolving obstruction is associated with improved survival, emphasizing the need for careful patient selection. Less than a third of patients receiving chemotherapy can continue the treatment once the colon obstruction has resolved. Future prospective studies should evaluate not only clinical outcomes but also quality of life and shared decision-making in palliative care settings.

## Data Availability

The authors confirm data transparency. No datasets were generated or analyzed during the current study.

## Abbreviations

SEMS: Self-expandable metal stent
CRC: Colorectal cancer
ECOG: Eastern Cooperative Oncology Group
ESGE: European Society of Gastrointestinal Endoscopy
OS: Overall survival
